# Preventive effect of the flavonoid, quercetin, on hepatic cancer in rats via oxidant/antioxidant activity: molecular and histological evidences

**DOI:** 10.1186/1756-9966-28-80

**Published:** 2009-06-11

**Authors:** AlaaEddeen M Seufi, Safinz S Ibrahim, Tarek K Elmaghraby, Elsayed E Hafez

**Affiliations:** 1 Entomology Department, Faculty of Science, Cairo University, Giza, Egypt; 2 Biochemistry Department, Faculty of Pharmacy, Cairo University, Cairo, Egypt; 3 Radiation Biology Department, National Center for Radiation Research and Technology, Giza, Egypt; 4 Department of Molecular Plant Pathology, Agriculture Research and Development Institute, Mubarak City for Scientific Research and Technology Applications, Alexandria, Egypt

## Abstract

**Background:**

The incidence of hepatocellular carcinoma is increasing in many countries. The estimated number of new cases annually is over 500,000, and the yearly incidence comprises between 2.5 and 7% of patients with liver cirrhosis. The incidence varies between different geographic areas, being higher in developing areas; males are predominantly affected, with a 2:3 male/female ratio

**Methods:**

Experiments were designed to examine the effect of *N*-Nitrosodiethylamine (NDEA) as cancer-inducer compound and to confirm the preventive effect of the flavonoid quercetin on hepatocellular carcinoma in rats. Briefly, thirty six male albino rats of Wistar strain were divided into 3 groups: the 1^st ^group was administered NDEA alone (NDEA-treated), the 2^nd ^group was treated simultaneously with NDEA and quercetin (NDEA+Q) and the 3^rd ^group was used as control (CON). Randomly amplified polymorphic DNA polymerase chain reaction (RAPD-PCR) as well as *p53*-specifi PCR assays were employed to determine genomic difference between treated, and control animals. Histological confirmation as well as oxidant/antioxidant status of the liver tissue was done.

**Results:**

RAPD analysis of liver samples generated 8 monomorphic bands and 22 polymorphic bands in a total of 30-banded RAPD patterns. Cluster analysis and statistical analyses of RAPD data resulted in grouping control and NDEA+Q samples in the same group with 80% similarity cut-off value. NDEA-treated samples were clustered in a separate group. Specific PCR assay for polymorphism of *P*^53 ^gene revealed a uniform pattern of allele separation in both control and NDEA+Q samples. Quercetin anticancer effect was exhibited in significant decrease of oxidative stress and significant decrease of antioxidant activity. Histopathological studies showed normal liver histology of the NDEA+Q samples. Meanwhile, several cancer-induced features were clearly observable in NDEA-treated samples.

**Conclusion:**

This paper demonstrated that preventive effect of quercetin on hepatocarcinoma in rats by RAPD-PCR, tracing the effect on *p53 *gene and by histopathological evidence. Hereby, it was proved that quercetin exerted its preventive effect via decreased oxidative stress and decreased antioxidant activity.

## Background

The incidence of hepatocellular carcinoma is increasing in many countries. The estimated number of new cases annually is over 500,000, and the yearly incidence comprises between 2.5 and 7% of patients with liver cirrhosis. The incidence varies between different geographic areas, being higher in developing areas; males are predominantly affected, with a 2:3 male/female ratio [1]. Malignant transformation of cell is due to the progressive accumulation of mutations, stable nonmutational (epigenetic) alterations in gene expression and/or gene product (protein) function [2]. Chemical carcinogens could be classified as genotoxic and nongenotoxic [3]. Although nongenotoxic carcinogen is not mutagenic, it may stimulate cell proliferation, inhibit apoptosis, increase inflammation, and/or induce stable or transient epigenetic changes in critical genes of terminally proliferating cells [3]. Nitrosamines are known as precarcinogens capable of inducing tumors in different animal species and are suspected of being involved in some human tumors [4]. *N*-Nitrosodiethylamine (NDEA), which is present in the environment [5] and in tobacco smoke, and is also synthesized endogenously [6], induces tumors in all species tested so far [7]. In contrast, Andrzejewski *et al*. [8] postulated that NDEA is epigenetic.

The antitumor effects of plant flavonoids have been reported to induce cell growth inhibition and apoptosis in a variety of cancer cells [9]. Quercetin, a ubiquitous bioactive flavonoid, can inhibit the proliferation of cancer cells [10,11]. It has been shown that quercetin treatment caused cell cycle arrests such as G_2_/M arrest or G_1 _arrest in different cell types [10,12]. Moreover, quercetin-mediated apoptosis may result from the induction of stress proteins, disruption of microtubules and mitochondrial, release of cytochrome *c*, and activation of caspases [11,13,14]. Li et al. [15] suggested that alpha methylacyl-coenzyme A racemase (AMACR) staining may serve as a useful marker for the differential diagnosis of well-differentiated HCC from HCA. Increased AMACR expression and its association with tumor venous invasion suggest that AMACR may play a role in HCC development and progression.

Lipid peroxidation, initiated in the presence of hydroxy radicals resulting in the production of malondialdehyde (MDA), directly produces oxidative stress [16]. Glutathione (GSH) is a key player in reduction processes in the cell. It also plays a role in reduction of NTPs to dNTPs and in detoxification of endogenous and exogenous compounds, serves as a cofactor for various enzymes, stores and transports cysteine, and may be involved in cell cycle regulation and thermotolerance [17]. Glutathione reductase (GR) is a gene encoding for an enzyme which reduces glutathione disulfide (GSSG) to the sulfhydryl form GSH, which is an important cellular antioxidant [18,19]. Glutathione peroxidase (GPX) is a general name of enzyme family with peroxidase activity whose main biological role is to protect the organism from oxidative damage. The biochemical function of glutathione peroxidase is to reduce lipid hydroperoxides to their corresponding alcohols and to reduce free hydrogen peroxide to water [18,19].

The main objectives of the present work were to examine the effect of NDEA as cancer-inducer compound and to confirm and throw light on the preventive effect of the flavonoid quercetin on hepatocellular carcinoma in rats. However, these issues are still debatable.

## Methods

### Animals and drugs

A total of 36 male albino rats of Wistar strain (170–200 g each), obtained from the central animal house of Faculty of Pharmacy, Cairo University, Cairo, Egypt were used in the present study. Animals were kept in groups at constant nutritional and highly controlled conditions: 23 ± 1°C temperature, 60 ± 10% RH and 12 L: 12 D photoperiod throughout the experimental period. The experimental protocols were approved by the Ethical Committee of Cairo University.

NDEA as carcinogenic material and the flavonoid quercetin, enzymes and coenzymes were obtained from Sigma-Aldrich Co. (St. Louis, Missouri, USA). Other chemicals were from Analar grade. NDEA was dissolved in saline (8 mg/1 ml vehicle). Quercetin was suspended in 0.5% carboxymethyl cellulose (20 mg/1 ml vehicle).

### Induction of liver carcinogenesis

Induction of liver carcinogenesis was carried out according the following protocol: each rat received an oral dose of 20 mg/kg (NDEA/weight), for 9 weeks (5 days/week) followed by another oral dose of 10 mg/kg (NDEA/weight) for 6 weeks (5 days/week).

### Experimental groups

Rats were acclimatized for 4 days before carrying out the experimental work. Animals were divided into 3 groups: the 1^st ^group (14 animals) was treated with NDEA for 15 weeks as detailed above and designated as (NDEA-treated), the 2^nd ^group (12 animals) was treated simultaneously with NDEA (20 mg/kg for 9 weeks followed by 10 mg/kg for 6 weeks) and Quercetin in a dose of 200 mg/kg daily, for 15 weeks as detailed above, the 3^rd ^group of rats (10 animals) was used as control (oral dose of saline was administered). At the end of the experimental period, rats were food-deprived overnight and were killed by cervical decapitation. The liver was immediately excised, rinsed with ice-cold saline and blotted dry and accurately weighed. A small portion of liver was fixed in 10% formal-saline for the histopathological studies.

### DNA extraction and amplification of RAPD markers

Genomic DNA was extracted from liver samples using Wizard Genomic DNA Purification kit (Promega, Madison, USA) following the manufacturer's instructions. DNA was visualized on a 0.7% agarose gel. Quality and concentration of DNA were determined spectrophotometrically. Three random primers were used to study the genetic difference between the examined animals. The primers used in this study are listed in Table 1. Optimization of PCR conditions for ultimate discriminatory power was achieved. RAPD-PCR was carried out in a 25 μl total reaction volume containing 2.5 μl 10× buffer, 0.2 mM dNT'Ps, 100 pmol primer, 2 U *Taq *DNA polymerase, 3.0 mM MgCl_2_, 50 ng DNA template and nuclease-free water. The amplification program used was 4 min at 94°C (hot start), 1 min at 94°C, 1 min at 30°C and 1 min at 72°C for 36 cycles followed by one cycle of 72°C for 10 min. PCR amplification was carried out in a DNA thermal cycler (Model 380 A, Applied Biosystems, CA, USA). PCR products were visualized on 2% agarose gel.

**Table 1:** Arbitrary primer sequences used in this study

Primer name	Primer sequence
EZ	5'-GCATCACAGACCTGTTATTGCCTC-3'

*Chi*^15^	5'-GGYGGYTGGAATGARGG-3'

*P*^53 ^F	5'-CATCGAATTCTGGAAACTTTCCACTTGAT-3'

*P*^53 ^R	5'GTAGGAATTCGTCCCAAGCAATGGATGAT-3'

### Specific PCR assay for polymorphism of *p*^53 ^gene

For the *p53 *PCR, DNA of control, hepatic carcinoma and quercetin-treated samples was used up for the *p53*-specific PCR assays. A primer set (Forward: 5'-CAT CGA ATT CTG GAA ACT TTC CAC TTG AT-3' and Reverse: 5'-GTA GGA ATT CGT CCC AAG CAA TGG ATG AT-3') was used for detection of *p53 *sequence. The PCR conditions were as follows: denaturation at 94°C for 5 minutes, then 35 cycles with denaturation at 94°C for 1 minute, annealing at 48°C for 1 minutes and elongation at 72°C for 1 minute. In the last cycle, the elongation step was extended to 10 minutes. PCR product (300 bp) was separated in 2% agarose gel.

### Oxidant/antioxidant status of liver tissue

Accurately weighed pieces of liver tissue were treated differently to study the oxidant/antioxidant status of the liver. Two portions were used to prepare 10% homogenate in 1.15% KCl and 5% homogenate in 3% sulfosalicylic acid, centrifuged at 1000 ×g at 4°C for 20 minutes. Resulted supernatants were used for the assay of malondialdehyde (MDA) as described by Yoshioka *et al*. [20] and glutathione (GSH) according to Srivastava and Beutler [21] levels, respectively. Portion of the liver was homogenized in Tris-sucrose buffer pH 7.4 (10% homogenate) and centrifuged at 15,000 ×g, at 4°C for 30 minutes, using Dupont-Sorvall Ultracentrifuge (USA), to isolate the cytosolic fraction. Cytosolic fraction was used for glutathione peroxidase (GPX) assay as described by Arthur and Boyne [22] and glutathione reductase (GR) according to Long and Carson [23]. Protein concentration of the above supernatant was estimated by the method of Lowry *et al*. [24].

### Histopathological examination of liver sections of the different groups

Slices of liver tissue were fixed in formal-saline, dehydrated in alcohol series and embedded in paraffin wax. Serial sections were made from each paraffin block, stained by eosin and hematoxlin dyes, and then submitted to histopathological examination under light microscope (Olympus Optical Corp., Tokyo, Japan).

### Statistical analyses

RAPD-PCR banding patterns of the liver samples were scored for the presence (1) or for absence (0) of each amplified band. All RAPD assays were repeated thrice and only the reproducible bands were scored. For considering a marker as polymorphic, the absence of an amplified product in at least one sample was used as a criterion. For genetic distance analysis, data sets were fed into the clustering program of SPSS (Version 14.0) and similarity matrix was determined using Jaccard's coefficient. Next, distance matrix (distance = 1 - similarity) was calculated. Based on similarity matrices using the unweighted pair group method analysis, STATISTICA program for Windows, 1995 (StatSoft, Inc., USA) was used to generate UPGMA dendrogram [25]. The Chi-square test was used to analyze the data obtained. Results of oxidant/antioxidant status were analyzed using one way analysis of variance (ANOVA) followed by Kruskal-Wallis test using SPSS software (Ver. 14.0). Differences were considered statistically significant if P < 0.05.

## Results

### RAPD analysis

**Figure 1:**
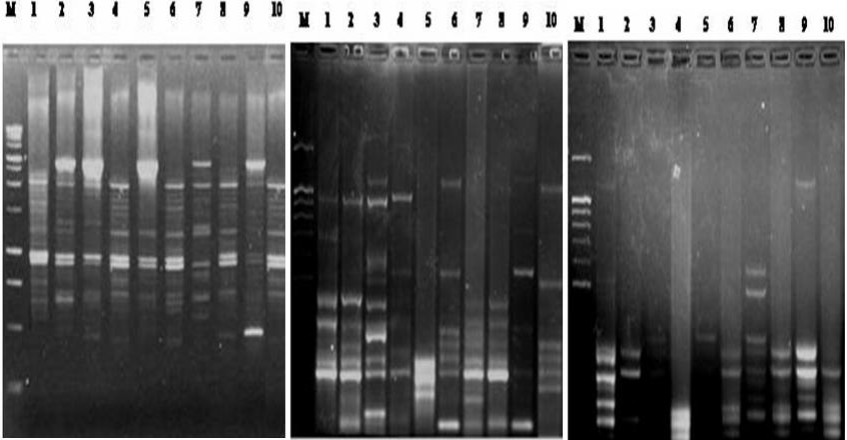
**Representative 2% agarose gels of RAPD-PCR patterns generated from 10 liver samples using three arbitrary primers: EZ: left, *Chi*^15^: middle and *P*^53 ^F: right**. Lane M: DNA marker 1 kb Ladder, lane 1: control animal, lanes 2–5: NDEA-treated animals and lanes 6–10: NDEA+Q-treated animals.

**Figure 2:**
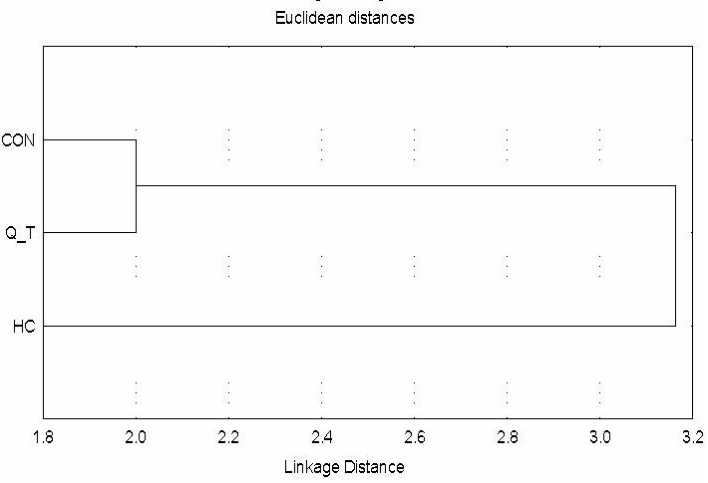
**A dendrogram constructed on the basis of similarity index among liver samples using the three RAPD primers**. CON: control, Q_T: NDEA+Q-treated and HC: NDEA-treated animals.

RAPD analysis of liver samples was carried out using four different primers. The results revealed that approximately 37 different banding patterns were obtained. Amplification with EZ primer generated 3 monomorphic bands and 6 polymorphic bands in a total of 9-banded RAPD patterns (Fig. 1). *P*^53 ^forward and reverse primers generated the same banding pattern: 2 monomorphic bands and 5 polymorphic bands in a total of 7-banded RAPD patterns. Whenever, *Chi*^15 ^primer generated one monomorphic band and 6 polymorphic bands in a total of 7-banded RAPD patterns (Fig. 1). A total of 30 distinct bands obtained were used for cluster analysis. The UPGMA dendrogram revealed that 80% similarity cut-off value gave two major clusters (RAPD genotypes: HC: NDEA-treated, Q_T: NDEA+Q group and CON: Control). NDEA+Q and control groups clustered in the same genotype while the NDEA-treated samples clustered in a separate genotype (Fig. 2). *Chi *square and Fisher's tests revealed that significant differences between both control and NDEA-treated and between NDEA-treated and NDEA+Q groups. However no significant difference between control and NDEA+Q groups was observed in case of primer *P*^53^.

### Specific PCR assay for polymorphism of *p*^53 ^gene

**Figure 3:**
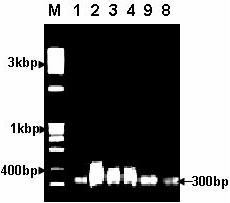
**PCR amplification of *p53 *exon from liver tissues**. Lane M: DNA marker, lane 1: control, lanes 2–4 NDEA-treated animals and lanes 8–9: NDEA+Q-treated animals.

Two oligonucleotide primers were designed to amplify 300 bp within the open reading frame (*orf*) of *p*^53 ^gene and were successfully used in PCR. PCR analysis of liver samples revealed a uniform pattern of allele separation in both control and NDEA+Q samples emphasizing the same results obtained by RAPD-PCR analysis (Fig. 3, lanes 1, 8 and 9). These results confirmed the preventive effect of the flavonoid quercetin on hepatocarcinoma in rats (Figs. 2 and 3).

### Oxidant/antioxidant status of liver tissue

The data presented in Table 2 show the oxidative stress (MDA concentration) and antioxidant activity (GSH, GR and GPX concentrations) of control, NDEA-treated and NDEA+Q treated liver tissues. MDA was studied as oxidative stress parameter while GSH, GR and GPX were estimated as indicators for antioxidant activity. Lipid peroxidation represented in MDA concentration showed significant increase (P < 0.001) in case of NDEA-treated rats in comparison to control (about 1.6 folds of control value). Treatment with quercetin (NDEA+Q) resulted in approximately normalization of MDA concentration (Table 2). Hepatic GSH content increased significantly (P < 0.01) in cases of both NDEA-treated and NDEA+Q group of rats in comparison to control group. Although treatment with quercetin (NDEA+Q) resulted in a significant decrease (P < 0.05) of hepatic GSH when compared to NDEA-treated rats, it still significantly higher (P < 0.01) than control GSH level (Table 2). NDEA-treated group exhibited significant increase (P < 0.01) in GR and GPX activities when compared to the control group. Treatment with quercetin (NDEA+Q) resulted in approximately normalization of GR and GPX activities, based on non-significant difference between NDEA+Q and control groups (Table 2).

**Table 2:** Effect of quercetin treatment on liver oxidant/antioxidant biomarkers in NDEA-induced liver carcinogenesis in rats

**Parameter**	**Control**	**NDEA-Treated group**	**NDEA+Q group**
MDA nmol/g liver	55.6 ± 3.41	90.4 ± 8.01^a^	60.8 ± 3.30^b^

GSH mg/g liver	1.5 ± 0.104	3.82 ± 0.149^a^	3.26 ± 0.088^ab^

GR nmol/mg protein/min	80.1 ± 2.53	101 ± 5.95^a^	83.6 ± 2.30^b^

GPX nmol/mg protein/min	324.36 ± 7.6	397.2 ± 13.16^a^	315.6 ± 6.09^b^

a. Significantly different from control.

b. Significantly different from NDEA-administered rats.

### Histopathological examination

**Figure 4:**
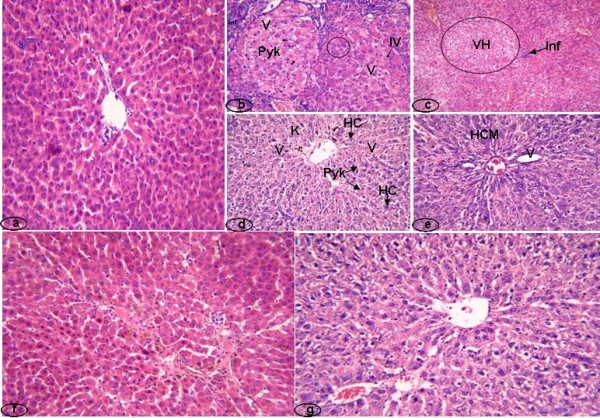
**Histopathological examination of animal livers**. a: control animals; b, c, d and e: animals treated with NDEA as cancer inducer; f and g: animals treated with NDEA+Q.

Hepatic histopathological features of control, NDEA-treated and NDEA+Q rats were illustrated in Fig. (4). Normal liver tissue showed hepatic lobule with normal architecture (Fig. 4a). Hepatic lobules were normal, each lobule consisted of normal hepatocytes arranged in hepatic strands, normal hepatic vein and each lobule contained blood vessels and bile ducts (Fig. 4a). No lipid droplets have been observed in the hepatocytic cytoplasm. No signs of blood congestion in blood vessels have been observed throughout the sections (Fig. 4a). Liver tissue of the NDEA-treated rats showed pleomorphism of nuclei, some cells exhibit multiple nucleoli (encircled), others are pyknotic (Pyk), some cells possess intranuclear vacuole (IV), some showed cytoplasmic vacuoles (V) and cellular infiltration (Inf) (Fig. 4b). Massive area of vacuolated hepatocytes (VH), cellular infiltration (Inf) and pyknotic nuclei were shown in Fig. (4c). Vacuolated cytoplasm (V), hyperchromatic nuclei (HC), pyknotic nuclei (Pyk) and numerous Kupffer cells (K) were seen in Fig. (4d). Hyperchromatic malignant nuclei (HCM) were exhibited in Fig. (4e). Liver tissue of the quercetin (NDEA+Q) treated rats showed normal hepatic lobule architecture (normal hepatocytes, hepatic vein, nuclei and blood vessels). Some bile droplets were observed in Fig. (4f). Fig. (4g) showed normal hepatocytes, hepatic vein, nuclei, bile ducts and blood vessels.

## Discussion

Hepatocellular carcinoma is the most frequent hepatic primary neoplasm. Its geographic distribution is inhomogeneous, with high, medium and low zones of incidence [26].

In the present study, RAPD, cluster and statistical analyses indicated the closer relation between control and NDEA+Q samples. Meanwhile, NDEA-treated samples were clustered in a separate group. These results were subsequently confirmed by specific PCR assay for polymorphism of *P*^53 ^gene which revealed a uniform pattern of allele separation in both control and NDEA+Q samples. To investigate how quercetin exerted its anticancer effect, oxidant/antioxidant status was tested. NDEA-treated samples exhibited allover higher oxidant/antioxidant status than control and NDEA+Q samples. Quercetin (NDEA+Q) succeeded in most cases to normalize the oxidant/antioxidant status of NDEA-treated samples. Moreover, histopathological confirmation showed normal liver histology of the NDEA+Q samples. Our results are agreeable with Lijinsky [4] and Bogovski and Bogovski, [7] who reported that NDEA is known as precarcinogen capable of inducing tumors in different animal species and are suspected of being involved in some human tumors [7]. Confirming results reported that administration of NDEA to rats resulted in lipid peroxidation (represented in higher MDA levels) and enhanced chemiluminescence in liver preneoplastic nodules, indicating the formation of activated oxygen species [27]. NDEA also produces 8-hydroxyguanine (8-OHG) [28], an indicator of oxidative damage to DNA (*P*^53 ^results) and the most abundant of more than 20 types of modifications produced under conditions of oxidative stress. This premutagenic DNA damage results in specific types of mutations and is likely to be involved in carcinogenesis. In contrast, Andrzejewski *et al*. [8] postulated that NDEA is an epigenetic chemical compound.

The antitumor effects of plant flavonoids have been reported to induce cell growth inhibition and apoptosis in a variety of cancer cells [9]. Quercetin, a ubiquitous bioactive flavonoid, can inhibit the proliferation of cancer cells [10,11]. It has been shown that quercetin treatment caused cell cycle arrests such as G_2_/M arrest or G_1 _arrest in different cell types [10,29]. Moreover, quercetin-mediated apoptosis may result from the induction of stress proteins, disruption of microtubules and mitochondrial, release of cytochrome *c*, and activation of caspases [11,30]. Granado-Serrano *et al*. [31] reported that quercetin may be a potential chemopreventive or therapeutic agent in hepatocarcinoma cells and further efforts to investigate these possibilities are needed. Specific *P*^53 ^gene PCR results may be contributed to the quercetin-mediated down regulation of mutant *P*^53 ^as reported by Avila *et al*. [32]. Contradictory results were reported by Chaumontet *et al*. [33] who reported the lack of tumor-promoting effects of the flavonoids. The oxidant/antioxidant status of liver samples illustrated that quercetin exerted its preventive effect through inhibition of lipid peroxidation to prevent oxidative DNA damage [28]. Consequently, the levels of GSH (a key player in reduction and detoxification processes) [17], GR (reduces GSSG to GSH which is an important cellular antioxidant) [18,19] and GPX (whose main biological role is to protect the organism from oxidative damage) [18,19] decreased significantly in NDEA+Q group. The higher concentration of GSH in NDEA+Q treated group than control could be attributed to the increase of GSSG-GSH transformation, by GR enzyme, to overcome the increased lipid peroxidation exerted by NDEA. Inhibitory hitopathological effect of quercetin looks like that reported in cyclo-oxygenase and phospholipase A_2 _inhibitors [34].

Conclusively, this paper demonstrated the carcinogenic effect of NDEA as well as the preventive effect of the flavonoid quercetin on hepatocarcinoma in rats by RAPD-PCR and by tracing the effect on *P*^53 ^gene. Oxidant/antioxidant results suggested that the eventual schedule of the cell is as follows: on treating rats with NDEA (NDEA-treated), lipid peroxidation increases (represented in high MDA concentration), GR enzyme succeeded in GSSG-GSH transformation (represented in high GSH concentration), GSH and GPX enzyme failed to exert antioxidant effect and could not protect organism against oxidative damage. Oxidative damage to DNA induced specific mutations (RAPD and *P*^53 ^PCR results) and these mutations are likely involved in carcinogenesis (histopathological evidence). In case of NDEA+Q group, lipid peroxidation inhibited (represented in normal MDA concentration), GR enzyme succeeded in GSSG-GSH transformation (represented in high GSH concentration), GSH and GPX enzyme exerted antioxidant effects and could protect organism against oxidative damage. DNA preserved its normal status (RAPD and *P*^53 ^PCR results) and hepatic lobule exhibited normal architecture. Hereby, it was proved that the mode of action by which quercetin exerted hepatic anticancer effect could be interpreted via oxidant/antioxidant status of the liver.

## Competing interests

The authors declare that they have no competing interests.

## Authors' contributions

AMS: Carried out the molecular genetic studies, participated in the design of the study, performed the statistical analysis, conceived of the study, and participated in its design and coordination.

SSI: Carried out the immunoassays, conceived of the study and participated in its design and coordination

TKE: Participated in the design of the study and performed the statistical analysis.

EEH: Carried out the molecular genetic studies, participated in the design of the study, performed the statistical analysis, conceived of the study, and participated in its design and coordination.
